# 

**Published:** 1888-01

**Authors:** 


### TABLE OF CONTENTS.

ORIGINAL AND SELECTED ARTICLES.

Renal abscess—nephrotomy—recovery, by Arch Dixon, M. D., Henderson, Ky. 1
Man as an object of zoology, by S. W. Stiles, M. D., Georgia. 3
Tyrotoxicon, its presence in cheese, ice cream and milk, and its relations to cholera-infantum, By V. C. Vaughan, M.D. 5
Three cases of sciatica, by J. W. Brown, of Silverton, Colorado. 9
A cure for Drunkenness. 11
Trichiniasis—nine cases, by G. W. Furey, M.D., Sunbury, Pa. 12
Temperature as a diagnostic and prognostic symptom, by Robert W. Martin, M. D., Chatham, Va. 16

ABSTRACTS AND GLEANINGS.

A substitute for iodoform. 17
The crowning achievement of ophthalmic surgery of the present century. 18
Indolent ulcers. 19
The treatment of acute bronchitis in children by antipyrin. 20
The pathogenesis of pterygium; cascara sagrada and its use in the treatment of constipation. 21
Treatment for carbuncle. 22
Indications for the use of nitro-glycerine; veratrum viride. 23
Injection for hemorrhoids. 24
Hints in the treatment of gonorrhœa; electricity as a pain killer; enlarged tonsils. 25
A valuable formula in menstrual irregularities; a new method of treating diseases of the skin locally. 26
Infantile constipation; phosphate of sodium hypodermically in phthisis. 27
The treatment of morphiomania; nitrate of potash as an emetic; ague-cake; to extract a tooth without pain. 28
Treatment of sciatica; cascara sagrada; iodide of sodium. 29
Biliary calculus; a large dose of croton oil; sensible; phosphate of lime. 30

SCIENTIFIC ITEMS.

Professor Tyndall on lightning conductors; a sea telephone. 31
A remarkable case of amnesia. 32

PRACTICAL NOTES AND FORMULÆ.

Insomnia; congestive chill. 33
Remedy for chronic malaria; for chafing infants; corrosive sublimate. 34
Aperient pill; treatment of acne; gastro-intestinal catarrh; constipation. 35

EDITORIALS AND MISCELLANEOUS.

Editorial brevities; shall we change the ℨ mark; a badge or distinction costume for physicians. 36
Our advertisers; the society of medicine; receipted. 37
Books and pamphlets received. 38
Special notes. 39

